# A numerical analysis of inclination and rectification of ramp-bridge piers adjacent to surcharge load in soft clay area

**DOI:** 10.1038/s41598-023-36737-6

**Published:** 2023-06-14

**Authors:** Boheng Shen, Dingtao Mao, Yong Ding, Lei Wang, Zhiyong Li

**Affiliations:** 1 Department of Civil Engineering, Ningbo University, Ningbo, 315211 China; 2 Ningbo Railway and West Hub Construction Center, Ningbo, 315042 China; 3 School of Mechanical Engineering and Mechanics, Ningbo University, Ningbo, 315211 China; 4 Ningbo Traffic Planning and Design Institute, Ningbo, 315199 China

**Keywords:** Engineering, Civil engineering, Mathematics and computing

## Abstract

The surrounding surcharge has an adverse impact on the service performance of buildings and bridges, and it can endanger their structural safety, especially in soft soil areas. As one case study, the inclination accident of an expressway ramp bridge and its rectification are investigated in this study. Through the three-dimensional (3D) finite element (FE) analysis of the overall structures composed of the bridge span, the pier, and the pile foundation, the whole process of the inclination by the adjacent dumped earth, partial recovery by the unloading, and the lateral pushing rectification of the bridge structure were simulated. The results show that the surcharge load leads to soil displacement near the bridge pile, and the pile-soil interaction leads to the pile deformation, which further causes the inclination of the pier, and the movement of the bridge span. The severity of the accident can be measured by the inclination of the piers and the opening widths of the bridge expansion joints. Due to the plastic deformation and drainage consolidation of the soft clay foundation under the surcharge load, the inclination of the piles and piers cannot be fully recovered after unloading. In order to capture these processes, the FE simulation was divided into three steps. First, the drainage consolidation of the soil foundation were identified by FE simulation and the field measurement of the recovery of the structure after unloading. Second, the effects of soil properties, the surcharge time and surcharge strength on the bridge inclination and the recovery capacity after unloading are discussed. Finally, the rectification of the bridge by lateral pushing was simulated, and the deformation and stress in the pier and pile were calculated to evaluate the safety of the structures. These analyses provided understanding towards the prevention of the bridge inclination under surcharge load, prediction of the recovery by the unloading, and the methods to reduce the residual deformation to meet the specifications.

## Introduction

With rapid development of urbanization and infrastructure construction in China, large domestic and construction waste have been produced. Due to the shortage of waste disposal land or the limitations due to the construction conditions, some waste is improperly placed near buildings or bridges that have been built or are under construction, resulting in the inclination of the piles and piers^1,2^, which, in turn, can results in the collapse of the structures^3,4^.

This problem is considered to be caused by the inclination of the pile under the lateral pressure of the soil layer under an adjacent surcharge load^5^. Previous studies of this issue, have included the field measurements^2,6–9^, centrifuge tests^10–12^, and numerical analyses^13–18^. These studies revealed that the lateral offset on the pile was caused by the adjacent unilateral surcharge, which resulted in the deflection of the superstructure such as bridge girders and high-rise buildings^19,20^. Ellis and Springman^21^ analyzed the interaction between the soil layer and the bridge abutment pile caused by the surcharge load beside the abutment, in which the plane strain finite element analysis was adopted, and the calculated results of the lateral earth pressure and deflection of the pile agreed with the result of the centrifuge test. Jeong et al.^22^ presented an investigation on the short- and long-term passive loading on the piles using the two dimensional elastoplastic-consolidation coupled FE analyses with large strain mode, and the results showed that passive pile loading was primarily affected by the magnitude of the surcharge load and degree of consolidation. Wang et al.^12^ performed four centrifuge model tests and numerical analyses to study the response of single pile subjected to lateral soil movement, and the results showed that it was necessary to consider the effect of consolidation on the pile-soil interaction. Li et al.^23^ conducted a series of field tests to examine the behavior of piles in the foundation with deep soft soils subjected to adjacent surcharge load, and a 3D FE model was established to study the factors affecting the consolidation of the soft soil. The results showed that the distance between the surcharge load and the foundation was more important than the magnitude, or the area of the load, and partial deformation of the piles recovered slowly after the unloading. Bian et al.^24^ investigated the response of piles foundations of the rail lines subjected to an adjacent surcharge load by centrifuge testing and 3D numerical modeling, The results showed that the lateral displacement of a pier, on the pile group, was dominated not only by the lateral displacement of the pile cap, but also by the inclination caused by the uneven settlement of the pile foundation.

Compared with theoretical studies, there are fewer case studies of the practical accidents. Chai et al.^3^ investigated the overturning failure of a 13-storey residential building in Shanghai, China, by the plane strain finite element analysis. The results indicated that failure of the building was probably initiated by the tensile crack in the reinforced concrete piles close to the adjacent excavation. And the excessive tensile stress in the piles was caused by the combination of the excavation on one side of the building and the temporarily dumped soil on the opposite side of the building. A simple but important lesson drawn from this accident was that uneven surcharge load should be avoided in and after building construction. Pan et al.^4^ studied a collapse accident of a bridge under the action of adjacent dumped earth in Hangzhou, China, by 3D finite element analysis, in which the Mohr–Coulomb model was used to study the elastoplastic properties of soil, and the pile-soil interaction was also considered. The results showed that the position and weight of the earth heap had a significant influence on the lateral displacement and internal force of the bridge. The excessive combined deformation of bending and torsion of the pile caused by the adjacent abandoned earth led to the failure of pile foundation and the collapse of the bridge.

The aforementioned studies provide the theories and numerical methods for studying the inclination of the pile foundation under surcharge load. There are, however, few practical case studies on the bridge pier deflection in soft soil area under adjacent surcharge load. The influence of superstructures, such as the bridge girders has not been considered, and theoretical or numerical analysis of the rectification of bridge pier has not been studied, either. A practical accident of the inclination of a ramp-bridge pier under adjacent surcharge load is studied in the present work. A superstructure-substructure-soil interaction model was established to simulate the pier inclination under surcharge load, recovery after unloading, and rectification of the residual deformation by the method of lateral pushing. The mechanism of the bridge deformation under adjacent surcharge load, such as the variations in the opening width of the bridge expansion joint and the inclination of the piles and piers, was studied by analyzing the lateral deformation of the soil foundation, the inclination of the piles and piers, and the displacement of bridge girder. By predicting the partial recovery of the bridge structure after unloading, and calculating the deformation and stress of the structure under lateral pushing load, the effectiveness of the rectification methods is discussed. Finally, the effect of the soil properties, the magnitude and lasting time of the surcharge load on the inclination and rectification of the bridge structure are discussed.

## Practical example

### Accident overview

**Figure 1:**
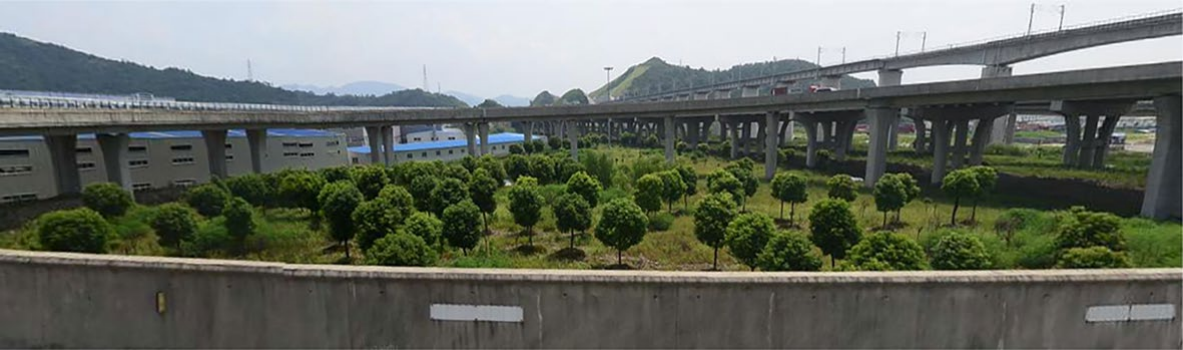
The curved ramp bridge before the dumping of the excavated earth in Xiaogang interchange of Loop Expressway in Ningbo, China.

The inclination accident occurred on the curved ramp-bridge of Xiaogang interchange of Loop Expressway in Ningbo, China (Fig. 1). The bridge was constructed in 2012. The superstructure of this ramp-bridge has five continuous girders, in which the first girder has four spans (30 m × 4), the second girder has three spans (20 m + 21 m + 20 m), the third girder has four spans (20 m × 4), the fourth girder has four spans (20 m + 21 m + 21 m + 20 m), and the last girder has four spans (20 m + 21 m + 21 m + 20 m). All the bridge piers are vase-shaped piers, under which the cast-in-place pile foundations with a top cap are used. All the bearings are seismic spherical bearings except for the fixed piers.

**Figure 2:**
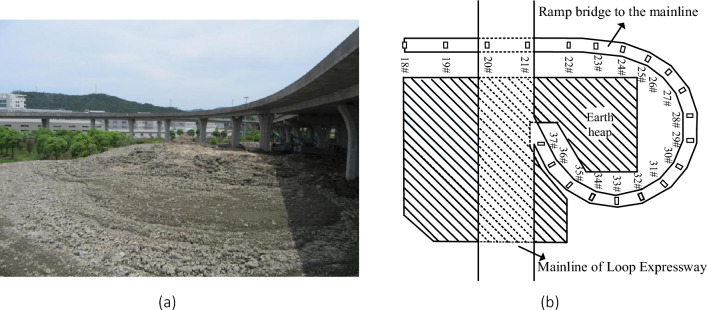
The curved ramp bridge after the dumping of the excavated earth: (**a**) photo of the bridge and the earth heap; (**b**) the plan view of the bridge and the earth heap.

On May 30, 2017, during the routine inspection of this bridge, it was found that the guardrails on both sides of the bridge deck were offset, and the opening widths of some expansion joints were too large, too small, or misaligned. Therefore, the substructure of the bridge was further inspected, and it was found that a large amount of excavated earth was dumped near the pier of the bridge (Fig. 2). The average height soil heap is about 3 m, and the maximum height is 6 m, and the area of surcharge load is about 17,000 m^2^.

### Measurements

After the inclination accident was found, the inclination of the piers and the opening widths of the expansion joints of the ramp bridge were measured in details, and the main results are as follows.

#### Pier inclination

**Figure 3:**
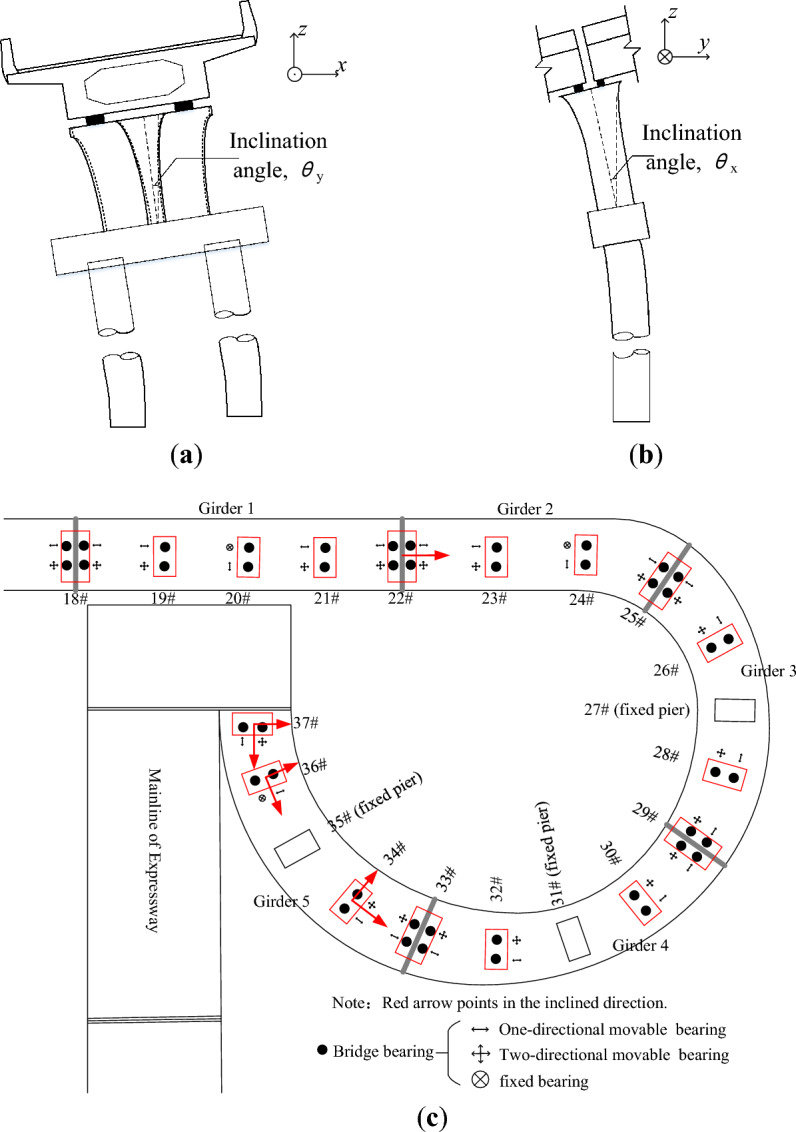
The inclination of the ramp-bridge under adjacent surcharge load: (**a**) inclination perpendicular to the bridge span; (**b**) inclination along the bridge span; (**c**) typical inclination of the bridge piers.

The measurements showed that some piers inclined under the adjacent surcharge load in Fig. 2, and 11 of 20 piers had an inclination greater than 3‰, which was defined as the maximum allowable inclination in Chinese standard^25^. The inclinations of typical piers are shown in Fig. 3 and Table 1. The piers were usually inclined towards the deposited earth, so that there is not only the inclination along the bridge span, but also that it was perpendicular to the bridge span.

**Table 1:** Measurements of the inclination.

No. of pier		Pier 22	Pier 34	Pier 35	Pier 36	Pier 37
Inclination (‰)	$\theta_{y}$	3.3	5.0	1.5	–	2.8
	$\theta_{x}$	1.1	4.2	1.3	0.7	1.8

#### The opening width of the bridge expansion joint

**Figure 4:**
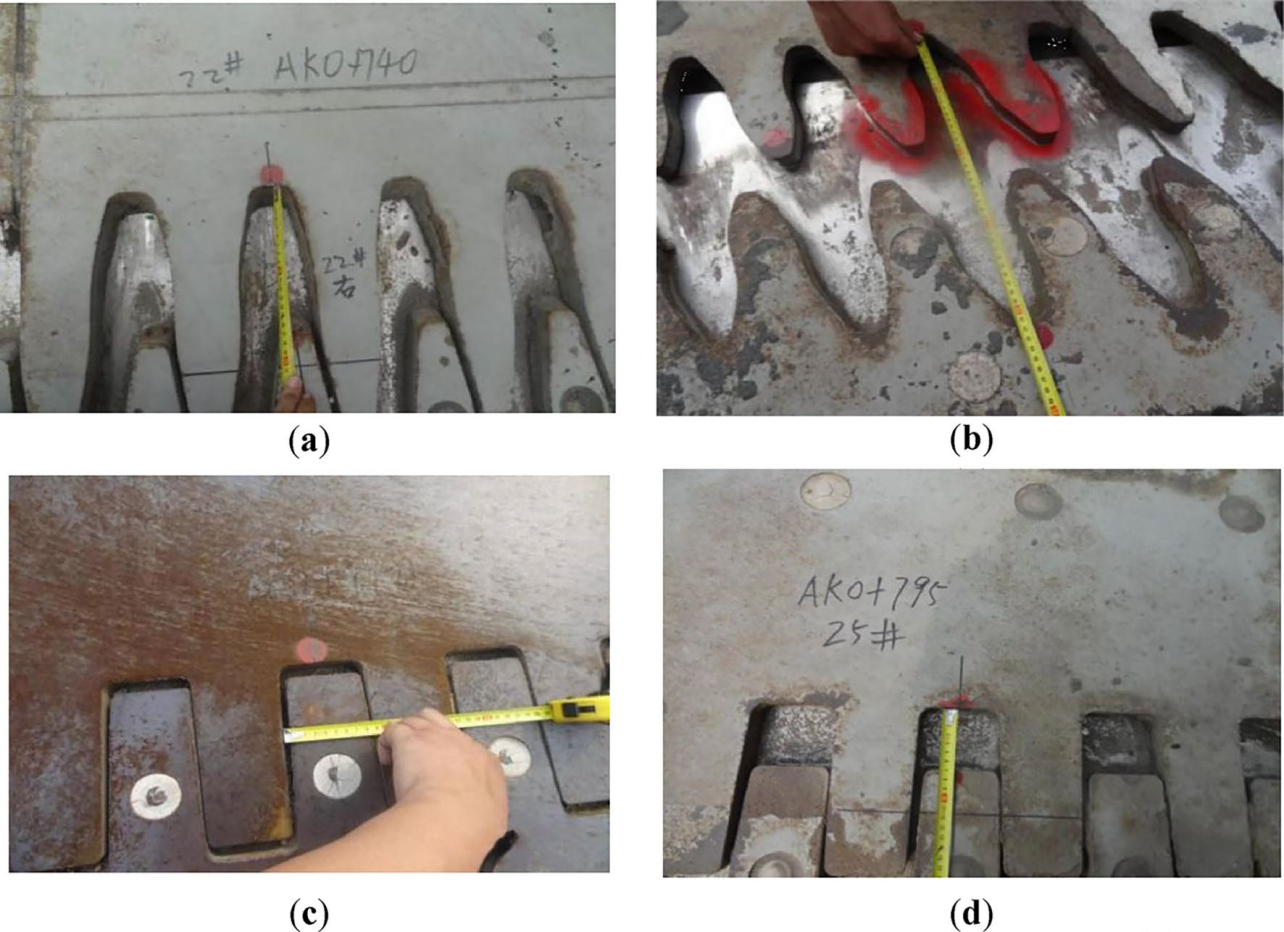
Disease of the expansion joints after the accident: (**a**) the expansion joint above pier 22 (over opening); (**b**) the expansion joint above pier 37 (over opening); (**c**) the expansion joint above pier 33 (insufficient opening); (**d**) the expansion joint above pier 25 (lateral extrusion).

Along with the inclination of the piers, the bridge spans move unevenly, which drives the bridge expansion joints to move unexpectedly, resulting in the loss of function of the expansion joints. The main diseases of the current bridge expansion joints after accident are shown in Fig. 4 and Table 2, in which $w$ is the opening width.

**Table 2:** Disease of the expansion joints after the accident.

Position	Above pier 22	Above pier 37	Above pier 33	Above pier 25
Disease	Over opening ($w$ = 17.5 cm)	Over opening ($w$ = 25.4 cm)	Insufficient opening	Lateral extrusion

The expansion joints used in the current ramp bridge are all 160-type bridge expansion joints with an opening width of 0–16 cm. It is because the longest continuous girder in the current ramp bridges is 120 m, the maximum expansion caused by changing temperatures, concrete shrinkage and creeping is about 11 cm^25^, so that the opening width of the current expansion joint is generally 2.5–13.5 cm. The opening widths of some expansion joints in this ramp bridge were, however, not in this range. For example, the opening widths of the expansion joints above piers 22, 37, and 33 were 17.5 cm, 25.4 cm, and nearly zero, respectively (Fig. 4a–c). The opening width of the expansion joint above piers 25 was 7 cm, which was in the acceptable range, but the lateral extrusion in it was severe (Fig. 4d).

### Rectification by the unloading

**Figure 5:**
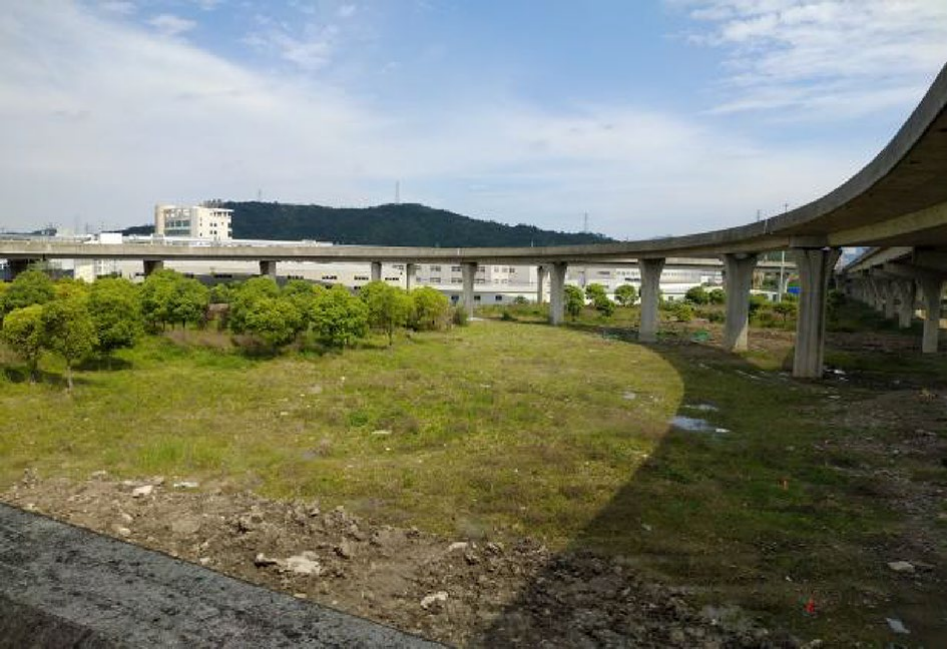
The bridge after unloading.

It is because the inclinations of 11 piers were greater than that specified in the standard, it affected the safety of the bridge, and therefore it must be corrected to restore the service performance of the bridge. Considering that the deposited earth near the bridge is the direct cause of the inclination of the piers, it was removed first. From June 1 to June 10, 2017, the deposited earth was removed by trucks, and the ramp bridge after this unloading is shown in Fig. 5. From July 2017 to November 2017, the inclinations of the piers were measured regularly, and they were stable after 5 months, which are shown in Table 3.

**Table 3:** Inclination of piers after 5 months of unloading.

No. of pier		18#	19#	20#	21#	22#	23#	24#	25#	26#	27#
Inclination (‰)	$\theta_{y}$	0.4	4.7	4.3	0.9	2.2	2.1	0.1	3.3	3.0	3.4
	$\theta_{x}$	1.0	3.6	4.9	0.9	0.6	3.9	3.7	0.4	1.3	2.5

No. of pier		28#	29#	30#	31#	32#	33#	34#	35#	36#	37#
Inclination (‰)	$\theta_{y}$	6.9	2.5	5.8	1.0	2.9	2.0	4.6	0.8	1.3	1.9
	$\theta_{x}$	4.7	4.4	6.4	1.3	0.8	1.9	4.2	1.0	1.2	1.2

As shown in Table 3, the inclination of the piers decreased partially after the unloading. For example, the inclination perpendicular to the bridge span (*θ *_*y*_) of pier 22 decreased from 3.3 to 2.2‰, and that of pier 37 decreased from 2.8 to 1.9‰. Yet, some piers still had the inclination greater than 3‰, such as pier 34.

Along with the piers, the girders also had some recovery after the unloading. As shown in Table 4, the opening width of the expansion joint above pier 37 decreased from 25.4 to 18.5 cm, and that of above pier 22 decreased from 17.5 to 16.5 cm. These two opening widths of the expansion joints were still beyond the allowable value, however.

**Table 4:** The opening widths of typical expansion joints (cm).

Position	Above pier 22	Above pier 37
Before unloading	17.5	25.4
After unloading	16.5	18.5

### Rectification by lateral pushing

After the unloading, there were still some piers with the inclination greater than 3 ‰ and several expansion joints with the opening width greater than the allowable value, and therefore it was found necessary to further rectify the bridge structure. According to the measurements after the accident, the plane line of the bridge span was close to that of the original design drawing, and the bridge deck remained flat and smooth when passing through the expansion joints. By so doing, only the pier inclination and girder displacement along the bridge span were rectified, and those perpendicular to the bridge span were neglected. By adopting this strategy, new diseases such as dislocation of expansion joints and irregularity of bridge span alignment caused by rectification can be avoided.

The method of lateral pushing was used to rectify the residual inclination and displacement of the ramp bridge. The displacements of the bridge spans were rectified first, and then the inclinations of the piers were rectified. As a case study, the rectification process of girder 1 in Fig. 3c is as follows.

#### The recovery of the girder position

**Figure 6:**
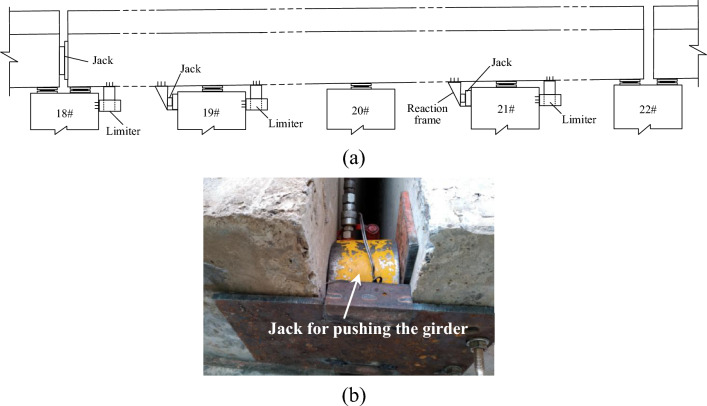
Recovery of girder 1 by jack pushing: (**a**) the position of the main and auxiliary jacks; (**b**) the main jack upon pier 20 for pushing the girder.

As shown in Fig. 6, because the opening width of expansion joint above pier 22 was greater than the acceptable value after accident, girder 1 in Fig. 3 was moved 4 cm towards pier 22. First, the steel safe limiters were installed on the top of the piers and the bottom of the girder to guide the girder moving along the bridge span and stop the movement perpendicular to the bridge span. And PTFE (Polytetrafluoroethylene) sheet was covered on the contact surface of the safe limit to reduce frictional resistance. Then the main jack was installed upon pier 18 and under the expansion joint (Fig. 6b), and the auxiliary jacks were set on the bottom of the girder near piers 19 and 21. Then the girder was pushed by the jacks to move 4 cm towards the direction of pier 22. During this pushing, the displacement of each pier was measured to prevent the damage to the piers or piles. It is because pier 20 was rigidly connected with girder 1.

#### The rectification of the piers

**Figure 7:**
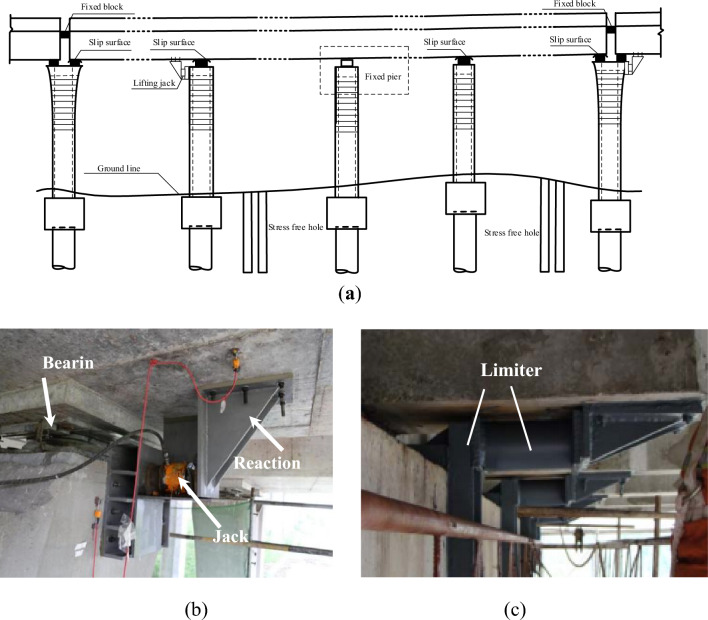
Recovery of piers by jack pushing: (**a**) the position of the jacks and slip surface; (**b**) the jack and reaction frame; (**c**) the steel limiter.

After the bridge girder was recovered, the piers with the inclination which exceeded 3 ‰ were rectified. As shown in Fig. 7a, the bridge girder was fixed first on both ends. Then the piers were rectified by jack pushing (Fig. 7b) one by one. After that, the jacks were replaced by the steel limiter (Fig. 7c) to keep the piers in the final position.

**Figure 8:**
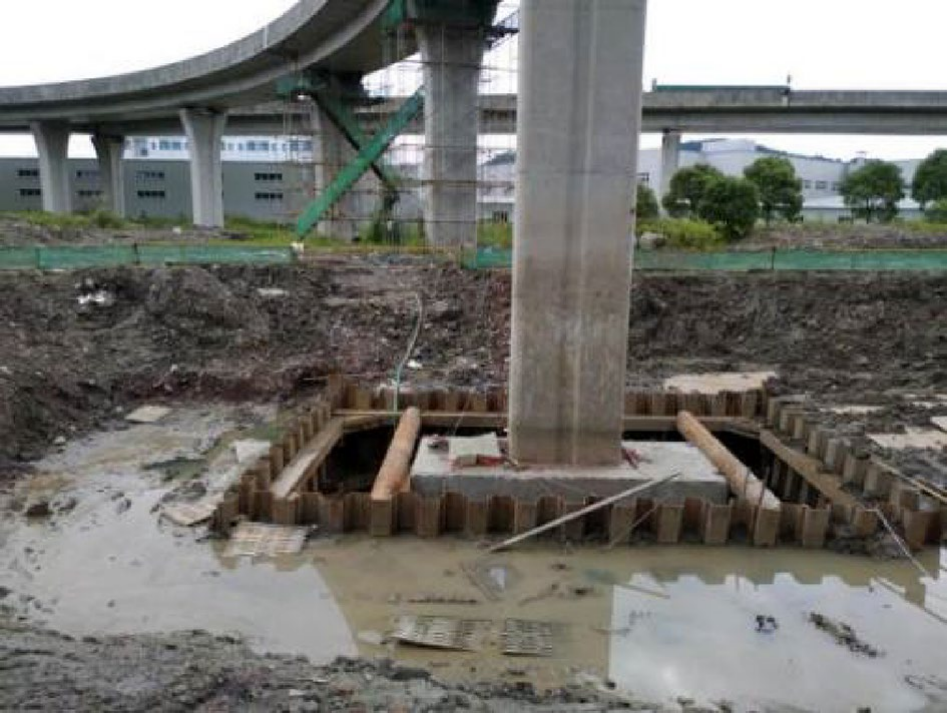
The excavated pile cap beneath the pier.

In order to make the pier to be rectified easily, the earth was excavated around the pile cap beneath the pier (Fig. 8), and some stress dissipation holes were drilled to reduce the lateral pressure of the soil.

After the above lateral pushing rectification, the inclination of the piers, and the opening widths of expansion joints were found to have recovered to acceptable values.

## Numerical analysis methods

**Figure 9:**
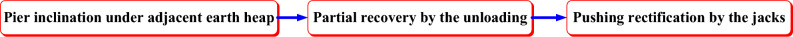
Flow diagram of 3D FEM analysis of the bridge including super- and sub-structure.

To ensure the safety of the above-mentioned rectification works, it was necessary to have the numerical analysis of the whole process of pier inclination under surcharge load, partial recovery by the unloading, and pushing rectification by the jacks. A 3D finite element method (FEM) is adopted for these analysis, and the flow diagram is shown in Fig. 9. When the bridge was under adjacent surcharge load, it is necessary to ascertain whether the pile foundation was damaged, and why the inclination of the structure occurred. When the adjacent surcharge load was moved away from the site, it was necessary to predict whether the inclination of the bridge can be eliminated after unloading. In the pushing rectification process, it was necessary to analyze the possible damage in the pile or the pier of the bridge, following which two, the maximum allowable displacement in the jack pushing was proposed.

### 3D numerical modeling

**Figure 10:**
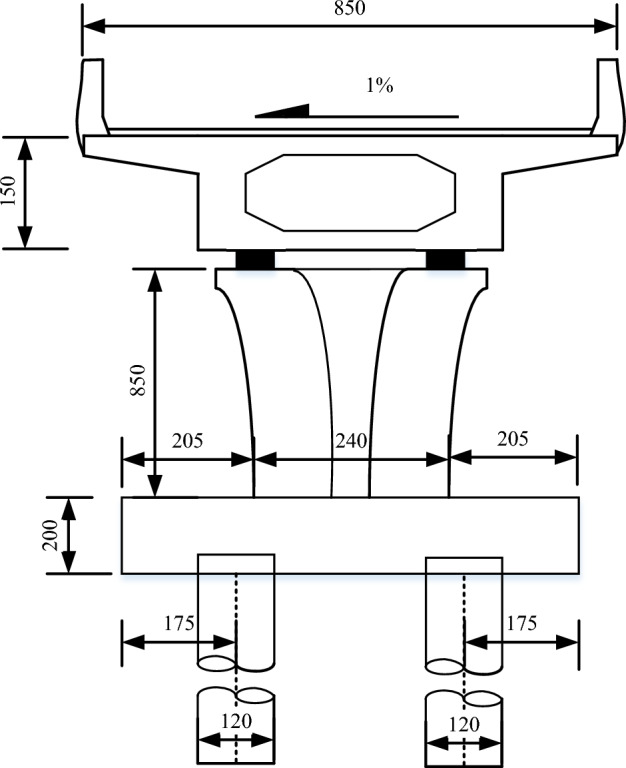
Typical section view of the bridge girder, the pier, and the pile foundation.

**Figure 11:**
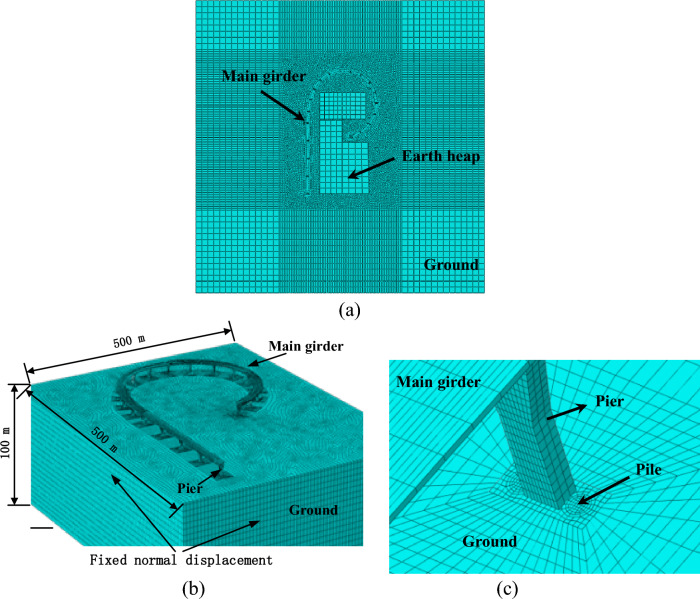
The 3D finite element model as used in this study: (**a**) the whole FEM model; (**b**) the bridge and the foundation; (**c**) the refined mesh of the pier and pile.

Considering the spatial characteristics of the curved ramp bridge and the surcharge load, 3D finite element model (FEM) was established for the super- and sub-structures of the bridge. The typical section of the bridge including the girders, piers, and pile foundations is shown in Fig. 10. The FEM model of the bridge and the foundation is shown in Fig. 11, which consists of 330,133 elements and 410,232 nodes. The bridge girder, the bearing, the pier, the pile, the soil layers surrounding the pile, and the surcharge load adjacent to the bridge are in the FEM model. The pile caps were tied with piles and piers. The contact was used to simulate the interaction between the soil and the piles with the frictional coefficient of 0.3^26^. Because the lateral deformation of piles is the concern in this study, and it is caused mainly by the pressure between the soil and piles, the variety of the frictional coefficient between piles and soil is not discussed. The effect of geometric nonlinearity was taken into account in the FEM analysis. The meshes of the studied areas and the frictional interfaces were refined. As shown in Fig. 11, the foundation size in the FEM model is 500 m × 500 m × 100 m, the normal displacement on the side of the foundation is fixed, the tangential displacement is free, the bottom of the foundation is fixed, and the shortest distance between the bridge structure and the model boundary is about 120 m. The soft soil layers in the foundation were 50 m deep with relatively rigid soil layers below. The piles of the bridge were all friction piles, and the maximum pile length was 80 m, so the soil foundation model of 100 m was deep enough. The FEM software, ABAQUS, was used in this simulation^27^. 3D element with the reduced integral (type C3D8R) was used for the bridge girders, the piers, the piles, the bearings and the earth heap. 3D element with pore pressure (type C3D8P) was used for the soil layers to evaluate the drainage consolidation.

### Material properties

The material properties of the bridge structure are shown in Table 5; the concrete was assumed to be linear elastic.

**Table 5:** Material properties of the bridge structure.

	Flexural-tensile strength (MPa)	Elastic modulus (GPa)	Poisson’s ratio	Density (kg/m^3^)
Girder concrete	5.5	34.5	0.2	2500
Pier concrete	4.5	30	0.2	2500
Pile concrete	4	28	0.2	2500

The bridge site, which has a deep soft soil layer in the upper part and a hard bearing layer in the lower part, is representative of the coastal areas of China. The stratification of the underground soil is shown in Table 6 according to the geological exploration of the site, and the mechanical parameters of the soil layers, recommended by Ye and Zhang^28^ were used.

**Table 6:** The material properties of the soil foundation.

No	Soil layer	Depth (m)	Density γ (kN/m^3^)	Void ratioe	Permeability coefficient k (m/s)
1	Silt	29	16.8	1.277	2.40 × 10^–8^
2	Muddy clay	21	17.3	1.244	2.00 × 10^–7^
3	Clay	10	17.2	1.082	3.67 × 10^–7^
4	Silty clay	40	18.7	0.826	4.85 × 10^–7^

Modified Cam Clay (MCC) model was adopted in elastoplastic simulation of the soil, which was proposed by Roscoe and Schofield^29^ and was subsequently modified by Burland and Roscoe (1969). The MCC model is, in general, suitable for consolidated clay and weakly over-consolidated clay, and it is also suitable for the numerical simulation of the surcharge-induced movement and drainage consolidation of the soft soil in Ningbo^30^. There are only three material parameters which need to be determined in this model, and all of them can be obtained by the conventional triaxial test. Therefore, the MCC model has become one of the most widely used models in the field of soil mechanics. The yield surface of MCC model is given as follows (Eq. 1):

1 $$\left( {p^{\prime} - \frac{{p^{\prime}_{0} }}{2}} \right)^{2} + \left( \frac{q}{M} \right)^{2} = \left( {\frac{{p^{\prime}_{0} }}{2}} \right)^{2}$$

where $p^{\prime}$ is mean effective stress, $q$ is deviator stress(shear stress), $p^{\prime}_{0}$ is preconsolidation pressure and $M$ is the slope of the critical-state line. By derivation, the incremental elastoplastic constitutive relationship of MCC model is shown in Eq. (2), which was used to form the elastoplastic matrix in finite element equations (Eq. 2):

2 $$\left\{ \begin{gathered} d\varepsilon_{V} \hfill \\ d\overline{\gamma } \hfill \\ \end{gathered} \right\} = \frac{\lambda - k}{\upsilon }\frac{2\eta }{{\left( {M^{2} + \eta^{2} } \right)}}\left( {\begin{array}{*{20}c} {\frac{\lambda }{\lambda - k}\frac{{M^{2} + \eta^{2} }}{2\eta }} & 1 \\ 1 & {\frac{2\eta }{{M^{2} - \eta^{2} }}} \\ \end{array} } \right)\left\{ \begin{gathered} \frac{dp^{\prime}}{{p^{\prime}}} \hfill \\ d\eta \hfill \\ \end{gathered} \right\}$$

where $d\varepsilon_{V}$ is the volumetric strain increment, $d\overline{\gamma }$ is the shear strain increment, $\lambda$ is the slope of the normal consolidation line, $k$ is the slope of the unloading–reloading line in the $\nu - \ln p^{\prime}$ plane, $\upsilon$ is the specific volume and $\eta$ is the stress ratio, $\eta = q/p^{\prime}$.^31^ The parameters in the above elastoplastic constitutive matrix, such as $k$, λ, and $M$, were taken directly from the laboratory experiments including the triaxial and consolidations test on soil samples in this area^32,33^, and they are shown in Table. 7.

**Table 7:** The parameters in MCC model of the soil.

No. of layer	Soil type	Depth (m)	$k$	*μ* (Poisson’s ratio)	*λ*	$M$	$p^{\prime}_{0} \,\left( {{\text{kPa}}} \right)$
1	Silt	29	0.04092	0.405	0.2236	0.8182	52.7
2	Muddy	21	0.03444	0.390	0.2107	0.8815	90.5
3	Clay	10	0.01860	0.368	0.1810	0.9624	109.0
4	Silty clay	40	0.01104	0.355	0.1083	0.9838	183.1

Because the earth heap has been unloaded, the practical material properties cannot be obtained. The Mohr–Coulomb model is used to describe the material properties of earth heap. The elastic modulus of the earth is assumed as 20 MPa, the density is 1800 kg/m^3^, the poisson's ratio is 0.35, the internal frictional angle is 40° and cohesive force is 10 kPa. Only the load effect of the earth heap is considered in this study, and top surface of the natural soil is set to drainage boundary with the pore pressure of zero.

### Coupled seepage-stress analysis of the underground soil

It is because the soil layers, where the pile foundation of this bridge is located, has a high water content, the coupling of the seepage field and the stress field should be considered in the structural analysis of the bridge under surcharge load. Further, the time-dependent soil drainage consolidation was analyzed. The direct coupling numerical method provided by ABAQUS based on the Biot consolidation theory (1941) was adopted to calculate the deformation and stress of bridge structure and underground soil under surcharge load. According to the principle of virtual work, the mechanical equilibrium equation of solid at time t is as follows (Eq. 3):

3 $$\int\limits_{V} {{{\varvec{\upsigma}}} \cdot \delta {{\varvec{\upvarepsilon}}}dV} = \int\limits_{S} {{\mathbf{f}}_{s} \cdot \delta {\mathbf{v}}dS} + \int\limits_{V} {{\mathbf{f}} \cdot \delta {\mathbf{v}}dV} + \int\limits_{V} {sn\rho_{f} {\mathbf{g}} \cdot \delta {\mathbf{v}}dV}$$

where ${{\varvec{\upsigma}}}$ is the stress, $\delta {{\varvec{\upvarepsilon}}}$ is the virtual strain, ${\mathbf{f}}$ is the force per unit volume, $\delta {\mathbf{v}}$ is the virtual displacement, ${\mathbf{f}}_{s}$ is the surface pressure, $s$ is saturation of soil, $n$ is the void ratio of soil, $\rho_{f}$ is the water density, and ${\mathbf{g}}$ is acceleration due to gravity, and $S$ and $V$ are area and volume of the structure.

The finite element equation of the structure was obtained by discretizing the above virtual work equation using the displacement finite element method. Fluids can flow through the finite element meshes, and the flow rate of the incoming fluid is equal to the rate of volume increase of the fluid according to the continuity equation, which is as follows (Eq. 4):

4 $$\frac{d}{dt}\left( {\int\limits_{V} {\frac{{\rho_{f} }}{{\rho_{f}^{0} }}} sndV} \right) + \int\limits_{S} {\frac{{\rho_{f} }}{{\rho_{f}^{0} }}sn{\mathbf{n}} \cdot {\mathbf{v}}_{f} } dS = 0$$

where t is the time, ${\mathbf{v}}_{f}$ is the average flow rate of the fluid, ${\mathbf{n}}$ is the outer normal vector on the boundary, $s$, and $\rho_{f}^{0}$ is the reference density of the fluid.

Using the Galerkin method, with displacement and pore water pressure as the nodal degrees of freedom, the above mechanical and seepage equations were spatially discretized, and the finite element mechanical equilibrium equations and seepage equations were obtained. This equation is as follows (Eq. 5):

5 $$\left[ K \right]\left\{ {\Delta \overline{\delta } } \right\} - \left[ L \right]\left\{ {\Delta \overline{p} } \right\} = \left\{ F \right\} - \left\{ I \right\}$$

where $\left[ K \right]$ is the stiffness matrix, $\left\{ {\Delta \overline{\delta } } \right\}$ is the displacement increment, $\left[ L \right]$ is the nodal force caused by the pore water pressure, $\left\{ {\Delta \overline{p} } \right\}$ is the pore water pressure increment, $\left\{ F \right\}$ is the external load, and $\left\{ I \right\}$ is the unbalanced force in the previous incremental iterative step.

The seepage equation is as follows (Eq. 6):

6 $$\left[ {\hat{B}} \right]^{T} \left\{ {\overline{v} } \right\} + \left[ {\hat{H}} \right]\left\{ {\overline{p} } \right\} = \left\{ Q \right\}$$

where $\left[ {\hat{B}} \right]$ is the volume change caused by the nodal displacement, $\left\{ {\overline{v} } \right\}$ is the derivative of the displacement increment ($\left\{ {\overline{\delta } } \right\}$) to the time, i.e., the velocity, $\left[ {\hat{H}} \right]$ is the seepage matrix, $\left\{ {\overline{p} } \right\}$ is the pore water pressure, and $\left\{ Q \right\}$ is the nodal flow.

As the time integral of the backward difference formula is introduced, the velocity is expressed as follows (Eq. 7):

7 $$\left\{ {\overline{v}} \right\}_{t + \Delta t} = \frac{1}{\Delta t}\left( {\left\{ {\overline{\delta } } \right\}_{t + \Delta t} - \left\{ {\overline{\delta } } \right\}_{t} } \right)$$

Thus, the seepage equation at the time of $t + \Delta t$ is expressed as follows (Eq. 8):

8 $$\left[ {\hat{B}} \right]^{T} \left\{ {\overline{\delta } } \right\}_{t + \Delta t} + \Delta t\left[ {\hat{H}} \right]\left\{ {\overline{p} } \right\}_{t + \Delta t} = \Delta t\left\{ Q \right\}_{t + \Delta t} + \left[ {\hat{B}} \right]^{T} \left\{ {\overline{\delta } } \right\}_{t}$$

Combined with the mechanical equilibrium equation, the coupled equations for soil seepage field and stress field are obtained as follows (Eqs. 9 and 10):

9 $$\left\{ \begin{gathered} \left[ K \right]\left\{ {\Delta \overline{\delta } } \right\} - \left[ L \right]\left\{ {\Delta \overline{p} } \right\} = \left\{ F \right\} - \left\{ I \right\} \hfill \\ \left[ B \right]^{T} \left\{ {\Delta \overline{\delta } } \right\} + \Delta t\left[ H \right]\left\{ {\Delta \overline{p} } \right\} = \left\{ R \right\} \hfill \\ \end{gathered} \right.$$

where,

10 $$\left\{ R \right\} = \Delta t\left[ {\left\{ Q \right\}_{t + \Delta t} - \left[ {\hat{B}} \right]^{T} \left\{ {\overline{v}} \right\}_{t} - \left[ {\hat{H}} \right]^{T} \left\{ {\overline{p} } \right\}_{t} } \right]$$

The transient stress field and seepage field of the underground soil were calculated by using Eq. (9).

## Calculation results and discussion

### Bridge inclination by the surcharge load and rectification by the unloading

In the calculation, the inclination of the super and lower structures of the bridge under the action of the surcharge load was first analyzed to evaluate whether the pile foundation in the underground soil was damaged, and which was used to provide a reference for structural maintenance or reinforcement in the next step. Then the effect of the unloading rectification was predicted, for the possible rectification by external forces.

It was because the original inclination of piers and the opening width of expansion joints were unknown, the exact values of the changes in the pier inclination and joint opening widths caused by the deposited soil could not be obtained. The pier inclination and the joint opening width before and after the unloading of the deposited soil were measured, however, so the recovery of the piers and expansion joints after unloading could be obtained to verify the reliability of the numerical calculations.

#### The condition of loading and unloading of surcharge load

**Figure 12:**
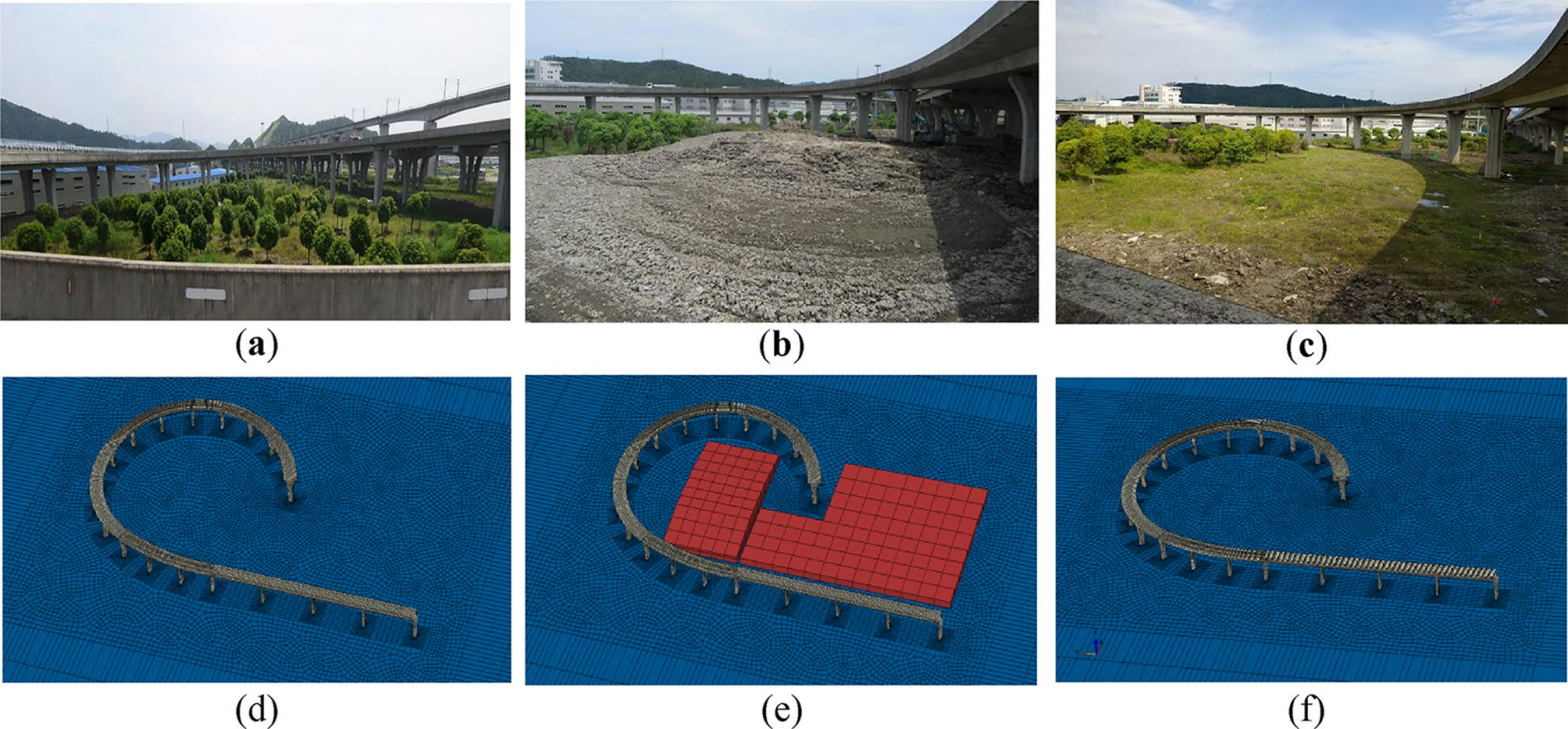
The working conditions: (**a**) before loading; (**b**) during loading; (**c**) after unloading; (**d**) FEM model before loading; (**e**) FEM model during loading; (**f**) FEM model after unloading.

The conditions of the bridge before loading, during loading, and after unloading are shown in Fig. 12. When the accident was found on May 30, 2017, the average height of the earth heap was about 3 m, the highest was about 6 m, and the area was about 17,000 m^2^. Unloading was carried out from June 1 to June 10, 2017, and the unloaded earth was about 7000 m^3^. The regular measurement of piers and expansion joints was done from June 1, 2017 to March 2018 when the rectification was finished.

Using the model and method proposed in this study, the deformation and stress of the bridge structure and the foundation during the loading and unloading processes were calculated. It is because the bridge is located in a soft soil area, the deposited earth will cause elastoplastic deformation and drainage consolidation of the underground soil. Drainage consolidation is a time-dependent process, and therefore the structural deformation is also a time-dependent process. In the calculation, it was necessary to determine the times of loading and unloading of the deposited earth.

Yet, it was not possible to find out when the earth was deposited. Therefore, another method was used to speculate this start time. First, the degree of soil consolidation was determined by the change of pier inclination during the unloading. Then, through the simulation of soil consolidation under the surcharge load, the loading time was speculated, and the starting time when the earth was deposited was determined. By using this method, the loading time of the deposited earth was inferred to be from March 13, 2017 to April 2, 2017, and it was assumed linearly increased from zero to the maximum value. Then this surcharge lasted for about 2 months until it was found and unloaded.

### Calculation results

#### Pier inclination

**Figure 13:**
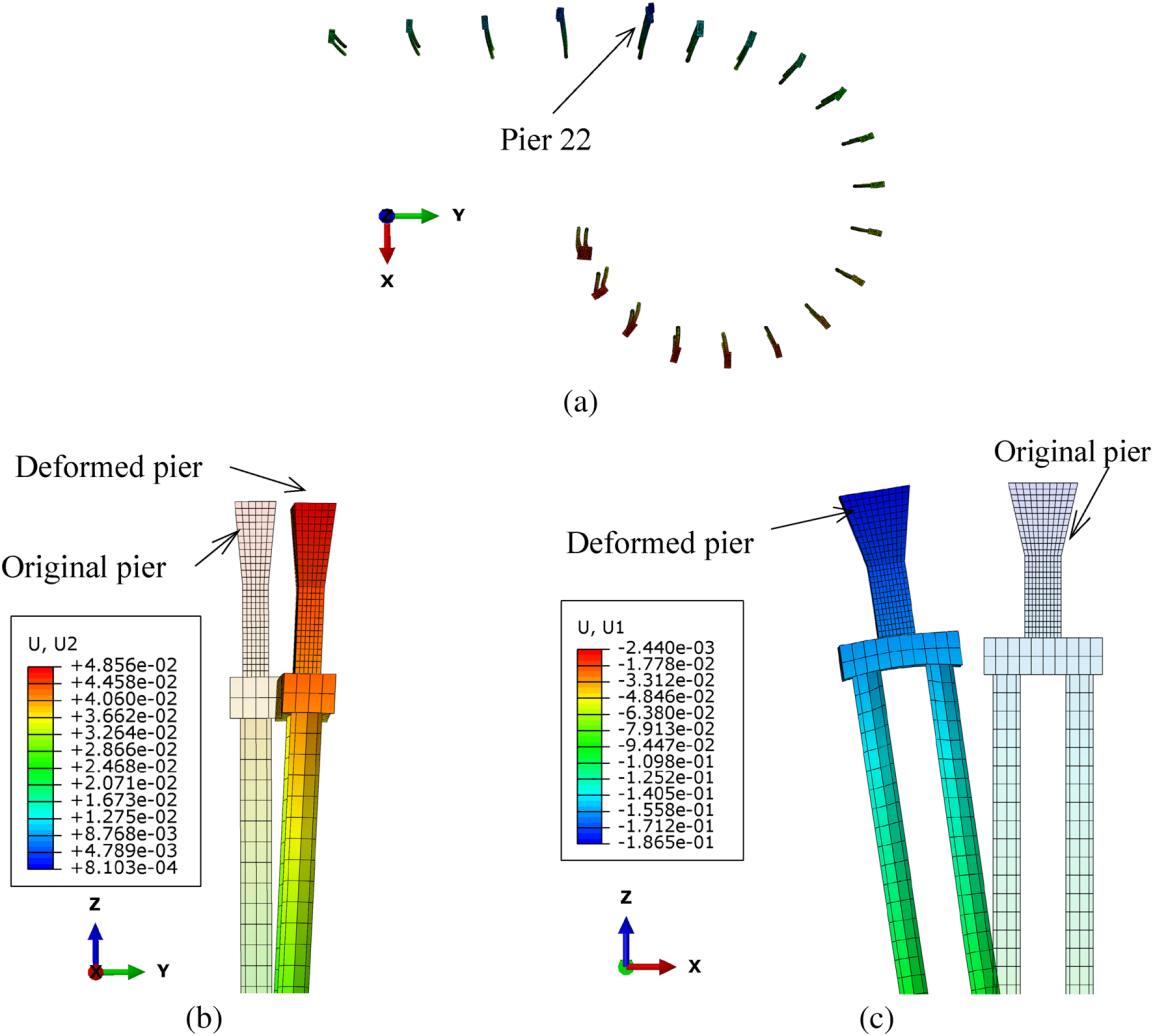
The inclination of the piers and piles under surcharge load (the deformation is magnified 50 times): (**a**) all the piers; (**b**) pier 22 along the bridge span; (**c**) pier 22 perpendicular to the bridge span (unit: m).

The calculated pier inclinations under the surcharge load are shown in Fig. 13. It indicates that all the piers are inclined towards the surcharge load, which is overall consistent with the measurements. The comparison between the calculations and the measurements of the typical pier inclinations are listed in Table 8, and the calculated and measured inclinations of piers have some differences. The reasons for this difference are as follows: (1) the initial inclination of the piers was assumed to be zero in the calculation, while that of less than 3 ‰ was allowed, and may exist in the actual bridge; (2) the magnitude and loading time of the surcharge in calculation may be different from those in practice; (3) the properties of soil in the calculations may be different from those of actual soil, such as whether it is completely saturated.

**Table 8:** Inclination *θ*_*y*_ of typical piers under surcharge (‰).

No. of pier	22#	34#	35#	37#
Calculation	3.02	1.52	2.14	2.48
Measurement	3.3	5.0	1.5	2.8

After the unloading, the calculated inclinations of typical piers are listed in Table 9, in which the results are overall consistent with the measurements. The inclination of the piers got reduced after the unloading, but some of these could not be restored to less than 3 ‰ as required by the standard^25^.

**Table 9:** Inclination *θ*_*y*_ of typical piers after unloading (‰).

No. of pier	22#	34#	35#	37#
Calculation	1.88	1.09	1.40	1.60
Measurement	2.2	4.6	0.8	1.9

**Figure 14:**
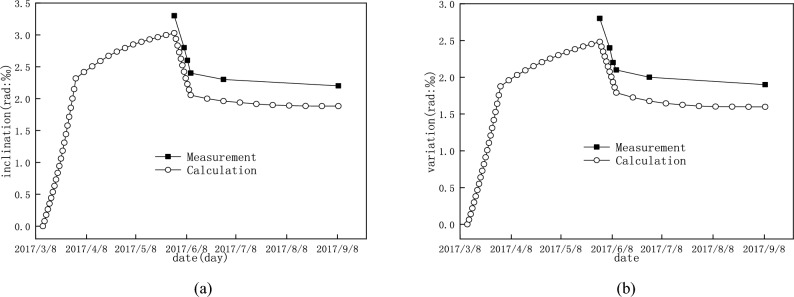
*θ*_*y*_ of typical piers when the initial inclination was assumed to be 0: (**a**) pier 22; (**b**) pier 37.

The history of the inclination of typical piers during and after unloading is shown in Fig. 14. It indicates that the numerical simulation can predict quite well the variation of the pier inclination. Therefore, on this basis, the initial inclination of the pier can also be speculated based on the numerical simulation. The accuracy of the calculated inclination of pier 22 is better than that of pier 37 due to the difference between the actual surcharge load and the assumed one in the FEM model, and the earth heap near pier 37 in the accident were less known.

#### The opening width of the expansion joint

**Figure 15:**
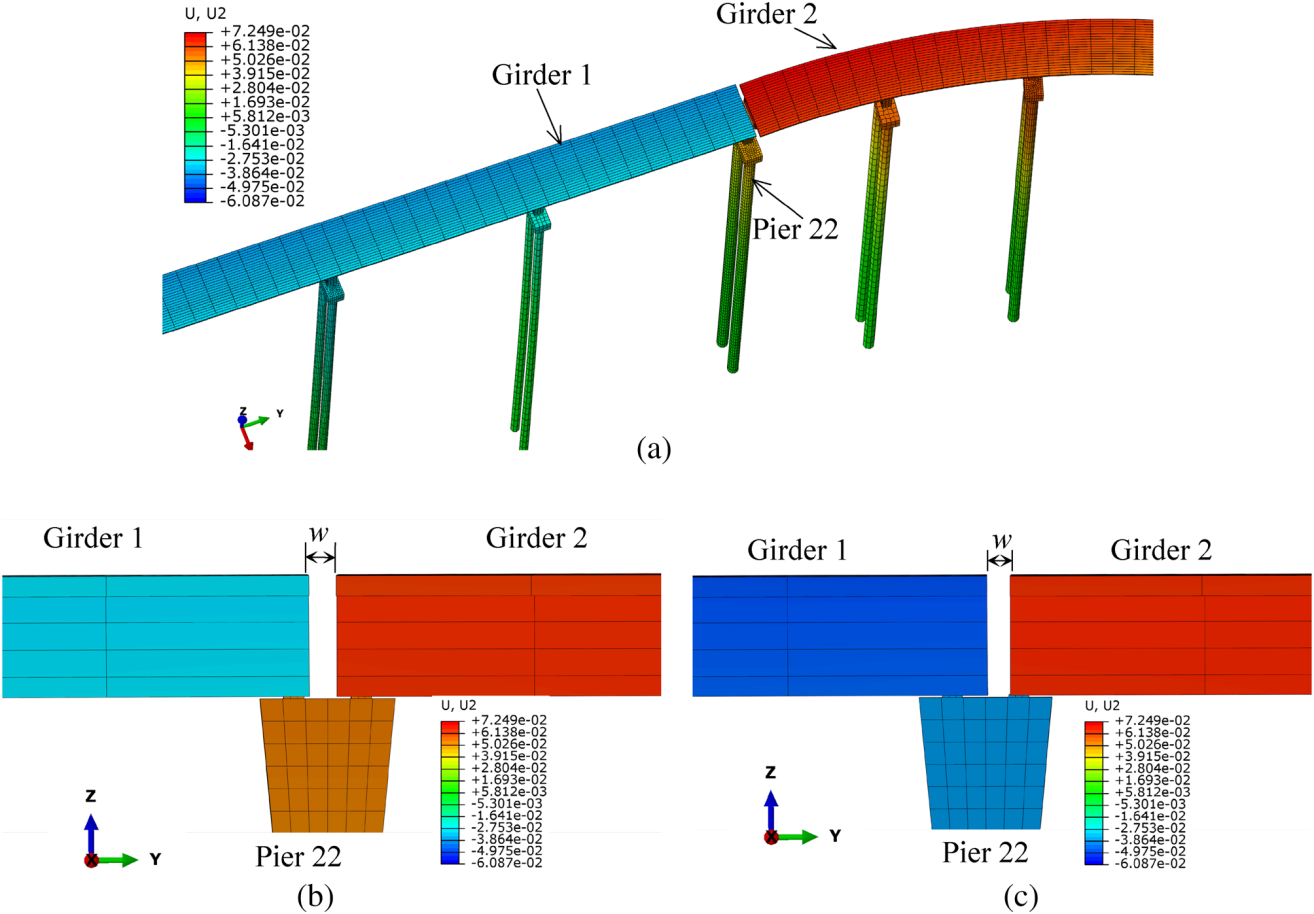
The variation of the opening width of the expansion joint above pier 22 (deformation is magnified 10 times) (**a**) the deformation of the girders and piers beside under surcharge load; (**b**) opening width of the expanson joint under surcharge load; (**c**) partial recovery of the opening width of expanson joint after unloading (unit: m).

The calculated variation of the opening width of typical bridge expansion joint is shown in Fig. 15. As shown in Fig. 15a, b, when the bridge structures are under the surcharge load, the deformation of girder 1 and girder 2 is opposite, which causes the opening width of the expansion joint, *w*, to become larger. As shown in Fig. 15c, after the earth heap is unloaded, the deformation of the girders is partially recovered, and the opening width of the expansion joint, *w*, becomes smaller.

**Figure 16:**
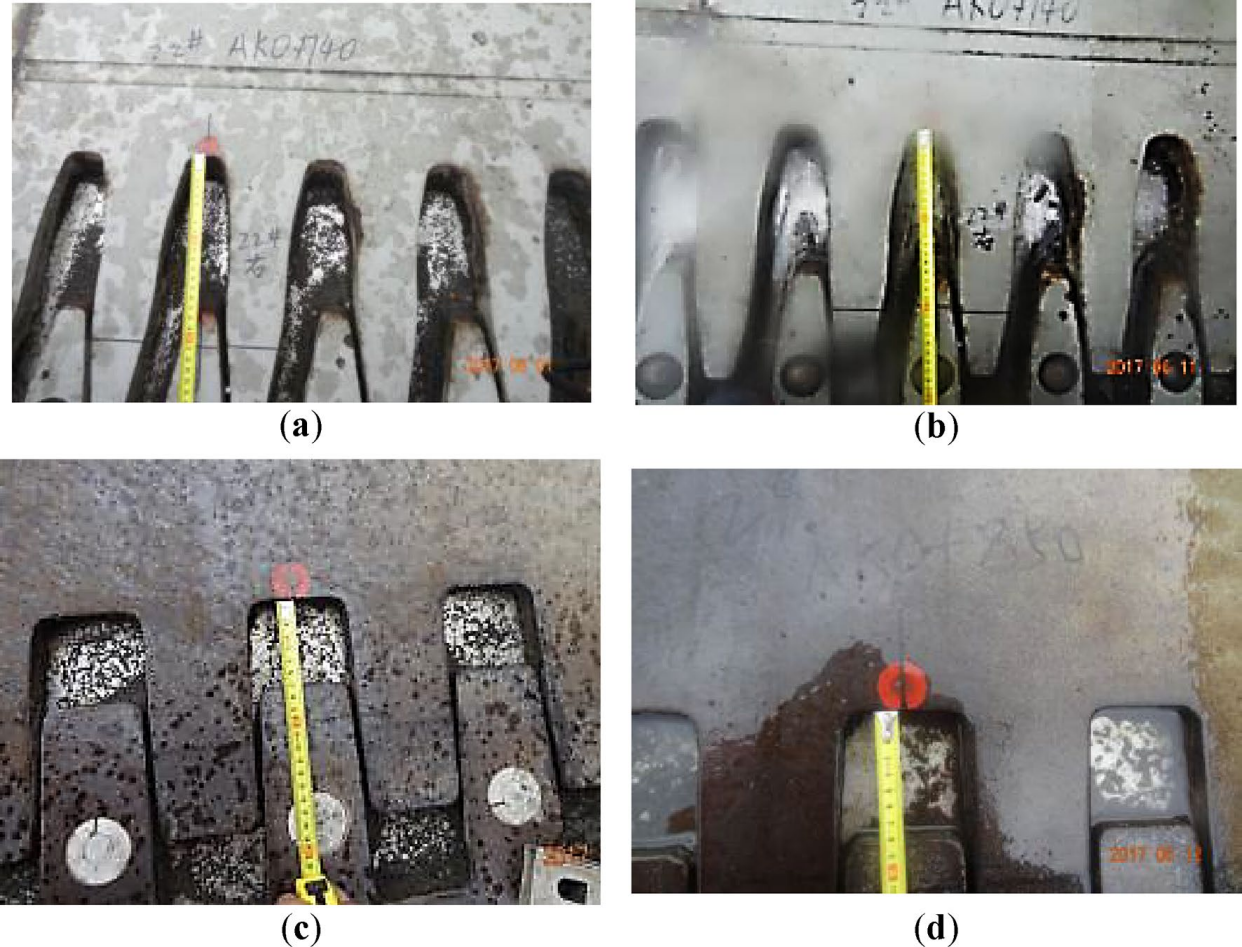
The opening width of the expansion joints: (**a**) above pier 22 before unloading; (**b**) above pier 22 after unloading; (**c**) above pier 29 before unloading; (**d**) above pier 29 after unloading.

The practical measurements also show that the loading and unloading of the surcharge have significant impacts on the opening width of some expansion joints on the ramp bridge. As shown in Fig. 16, after unloading, the opening width of the expansion joint above pier 22 decreased gradually by about 10 mm, and that above pier 29 increased gradually by about 18 mm.

**Figure 17:**
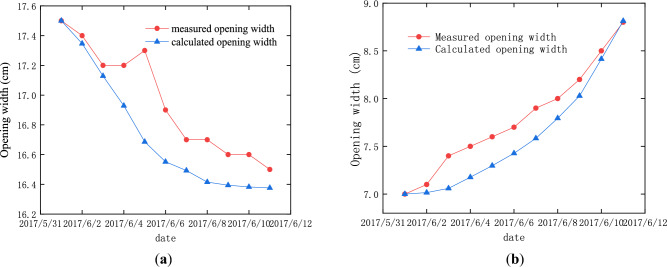
The variations in the opening width of the expansion joints in the process of unloading: (**a**) the expansion joint above pier 22; (**b**) the expansion joint above pier 29.

In order to compare the calculated result with the measurement, it is assumed that the initial expansion joint width from the calculation is same as that from measurement at the beginning of the earth heap unloading. The changes in the opening width of the expansion joints above pier 22 and pier 29 are shown in Fig. 17, which occurred within 12 days after the start of unloading. These results indicate that the change of the opening width of the expansion joint was predicted well by numerical simulation, and the maximum difference between the calculation and measurement was less than 5%. The calculation results also show that the surcharge loading was the main reason for the change of the opening width of the expansion joints. Further, the service performance of the expansion joints on the bridge deck can be used as an important indicator for the service performance of the bridge under the action of the adjacent surcharge load, and this indicator was easier to find because it is on the bridge deck.

#### Stress of the pile foundation

Due to the existence of expansion joints and elastic bearings, the movement of the bridge girder is relatively free, so the stress in the girder caused by the inclination of the pier is small, and the deformation can be measured. For the substructure, however, especially the pile foundation embedded in the soil layers, directly bears the earth pressure and is most affected by the surcharge load. Furthermore, the deformation and stress of the pile were difficult to be measured, so it was necessary to obtain the response of pile foundation under the surcharge load through theoretical analysis to judge whether it was damaged.

**Figure 18:**
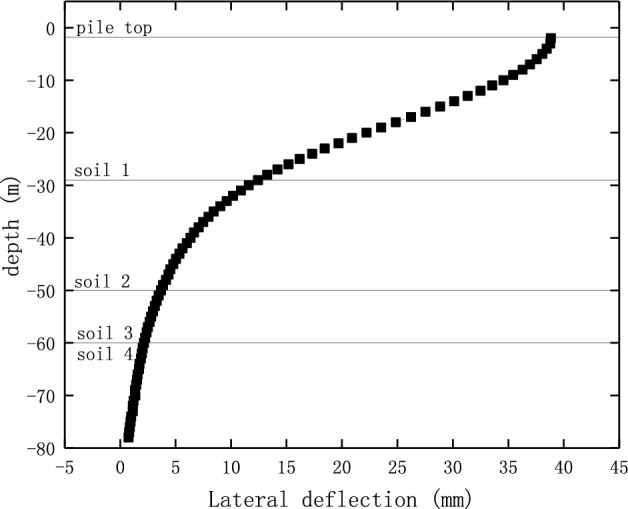
The lateral deflection of pile 22.

In order to simulate the pile-soil interaction accurately, a 3D solid element was used to simulate the pile structure. Also, the meshes of pile were refined to obtain the deformation and stress distribution accurately. Figure 18 shows the lateral deflection of pile 22 under the surcharge load, which is consistent with that of in previous studies^23,24^.

**Figure 19:**
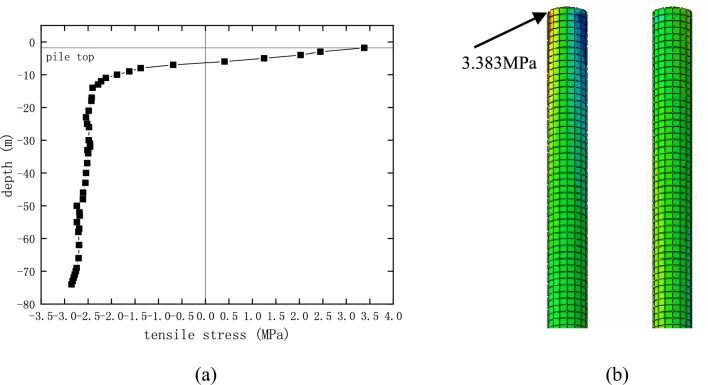
Tensile stress in pile 22: (**a**) the distribution of tensile stress in the pile; (**b**) the maximum tensile stress.

In general, the main failure of the concrete pile, under the influence of lateral earth pressure, is bending cracking, and thus it is necessary to calculate the stress in the pile. The calculated principal tensile stress of a typical pile beneath pier 22 is shown in Fig. 19. The calculations indicate that the underground pile was subjected to lateral earth pressure under the action of ground surcharge load, and the maximum principal tensile pressure was located at the top of the pile (Fig. 19b). The maximum tensile stresses in the piles of the ramp bridge are shown in Table 10. These stresses do not exceed the flexural-tensile strength of C25 concrete (4 MPa) used in the current bridge pile, and thus so it will not crack under the influence of surcharge beside the bridge.

**Table 10:** The maximum tensile stress in the piles beneath the piers.

No. of pier	18#	19#	20#	21#	22#	23#	24#	25#	26#	27#
Tensile stress (MPa)	2.489	1.537	3.163	3.799	3.383	2.734	2.545	2.204	0.815	1.413

No. of pier	28#	29#	30#	31#	32#	33#	34#	35#	36#	37#
Tensile stress (MPa)	0.910	1.559	1.298	1.139	1.135	2.300	1.829	3.104	1.215	3.624

#### Parametric analysis of loading and unloading condition

In the parametric analysis, the main factors, affecting the inclination and recovery of bridge structures, are the soil properties, and the duration and magnitude of the surcharge load, which are discussed in the following.

#### The effect of soil properties

In order to study the influence of soil properties on the inclination and recovery of the pier, soil layers with different properties can be selected for analysis^34^. As shown in Table 11, three types of soil layers with different hardness were selected in the present study.

**Table 11:** The types of soil layers used in the analysis.

Type 1			Type 2			Type 3		
No	Property	Depth (m)	No	Property	Depth (m)	No	Property	Depth (m)
1	Silt	29	1	Muddy clay	50	1	Clay	50
2	Muddy clay	21						
3	Clay	10	2	Silty clay	50	2	Silty clay	50
4	Silty clay	40						

**Figure 20:**
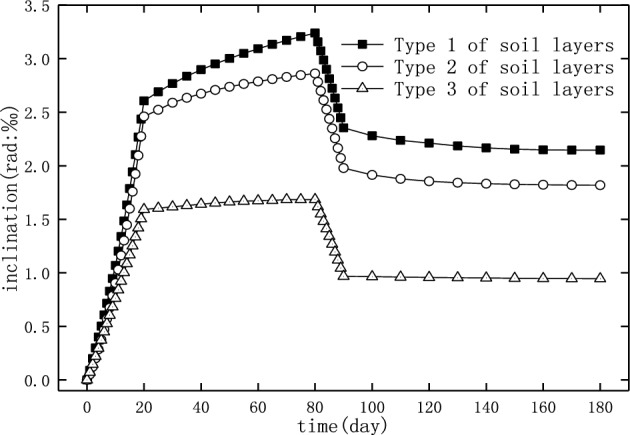
The inclination of pier 22 for the different soil layers.

The load in numerical simulation was similar to the practical loading and unloading of the deposited earth beside the bridge, that is to say, the surcharge load was increased from 0 to the maximum in 20 days, then maintain for 60 days, and then was removed in 10 days. Figure 20 shows the history of the inclination of pier 22 with this load in which the three types of soil layers (Table 11) were adopted, respectively. The results showed that higher the hardness and the lower the water content were of the foundation soil, the smaller was the inclination of the pier caused by the adjacent surcharge load, and higher was also the proportion of recovery after unloading. The reduction ratio of the pier inclination after unloading is 40.05%, 43.48%, and 53.4% for soil layers of type 1, type 2 and type 3, respectively. Therefore, when the soil layer was hard and the water content was low in the actual case, the inclination of the pier may have been reduced below the value allowed by the specification after unloading. Otherwise, further rectifications by external force should be carried out after unloading.

#### The effect of loading time

**Figure 21:**
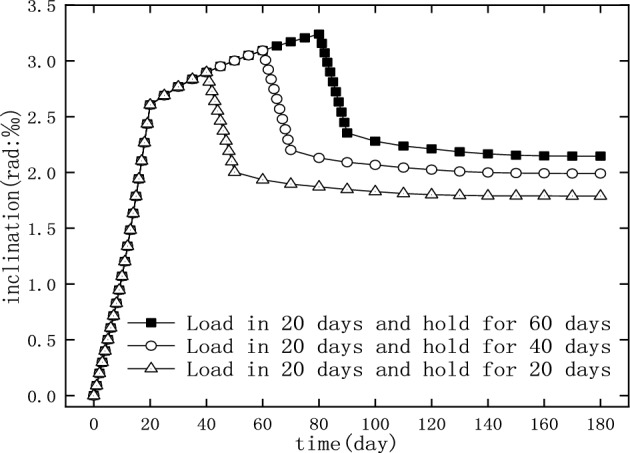
The inclination and recovery of the pier in different loading time.

In order to study the influence of loading time on the inclination and recovery of the piers, three different loading durations, namely 20, 40, and 60 days, were selected for the numerical simulations, and the results are shown in Fig. 21. The drainage consolidation of foundation soil is not immediately completed after the applying of earth heap. The drainage consolidation of soil is a long process, and the inclination of the pile slowly increases until it reaches stability^35^. In this process, the drainage consolidation of foundation soil did not reach a stable state, so the longer the loading time, the more serious was the drainage consolidation of the foundation soil, and greater was the inclination of the pier.

The recovery ratio of the pier inclination after unloading is shown in Table 12: with the increase of loading time, the recovery ratio decreases. Therefore, when the loading time was too long, the inclination of the pier, after the unloading, may not be able to recover to the acceptable value, and that further rectification might be needed.

**Table 12:** Recovery of inclination after unloading for different loading time.

Load duration time (in days)	20	40	60
Recovery ratio (%)	38.23	35.64	33.73

#### The effect of the surcharge magnitude

**Figure 22:**
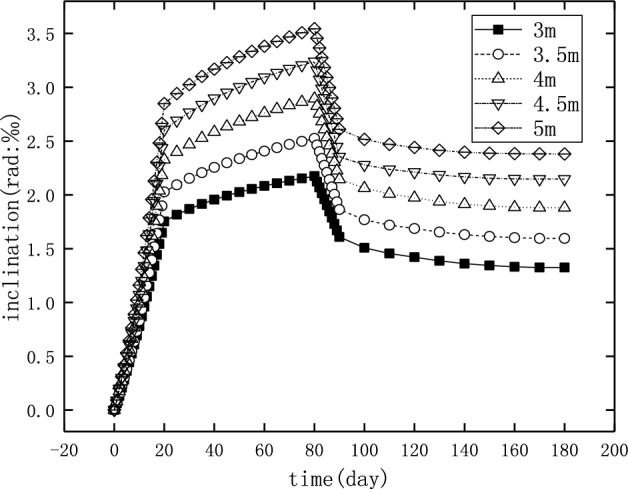
The inclination and recovery of the pier with different surcharge magnitude.

A previous study found that the surcharge strength was an important factor affecting the behavior of adjacent piles in soft soils (Hongquan, 2019). Therefore, three earth heaps with the height of 3 m, 4 m, and 5 m were modeled to investigate the effect of surcharge magnitude on the inclination of pier 22, and the results are shown in Fig. 22. It can be seen that the inclination of the pier increased with the increase of the loading strength. The recovery ratio of the pier inclination, after the unloading, is shown in Table 13. These results indicate that larger was the surcharge load, the smaller was the deformation that could be recovered after unloading.

**Table 13:** Recovery of inclination after unloading for different surcharge magnitude.

Height of earth heap (m)	3	3.5	4	4.5	5
Recovery ratio (%)	39.09	36.81	35.04	33.73	32.86

### Lateral pushing rectification of the girder and pier

#### Lateral pushing rectification of the girder

**Figure 23:**
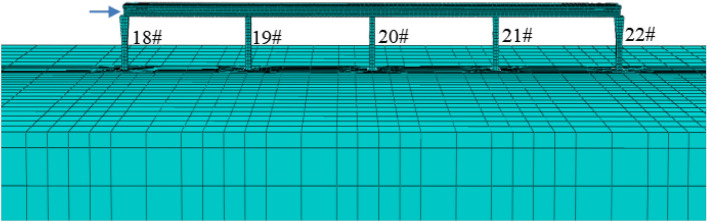
The finite element model of the lateral pushing of the girder.

The FEM model of lateral pushing of the bridge girder 1 is shown in Fig. 23. Because the opening width of the expansion joint on pier 22 is 16.5 cm after unloading, which exceeds the acceptable value of type 160 expansion joint, a lateral force was applied on the girder 1 above pier 18 to move it towards pier 22 to reduce the opening width of expansion joint above pier 22 (Fig. 23).

**Figure 24:**
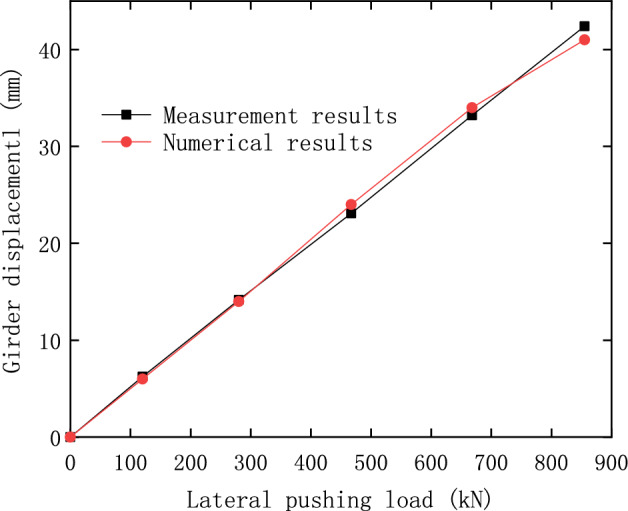
Movement of the girder in the lateral pushing rectification.

The comparison between the calculations and measurements of the lateral pushing process of the girder is shown in Fig. 24, in which the results reflect the relationship between displacement and the pushing force during the girder rectification.

**Figure 25:**
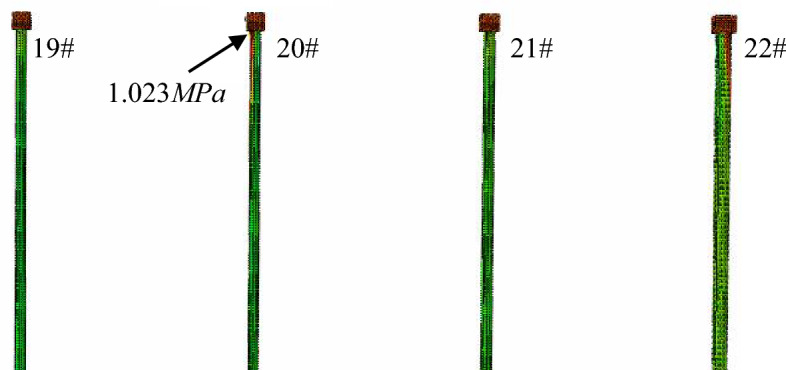
The maximum tensile stress of piles below girder 1 of the bridge.

It is because the girder is consolidated with pier 20, its displacement will cause the bending of the pier 20 the piles below. Therefore, it was deemed necessary to predict the tensile stress of piers and piles caused by such bending to prevent cracking damage. Figure 25 shows the calculated tensile stress in the pile foundations. The maximum tensile stress, 1.023 MPa, located at the pile below pier 20, is less than the tensile strength of concrete C25 used for the pile, 1.27 MPa. Therefore, this suggests that the substructure will not be damaged during jack pushing.

#### Lateral pushing rectification of the pier

**Figure 26:**
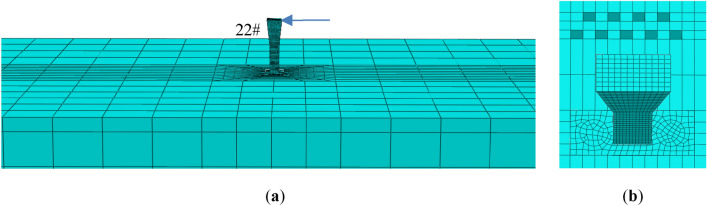
The FEM model of lateral pushing of the pier: (**a**) the whole model; (**b**) the pier and the stress dissipation hole.

After the girder pushing was completed, the pier with the inclination along the span which exceeded 3 ‰ was rectified, such as pier 22 in Fig. 26a. Using the reaction force provided by the main girder, the top of the pier was pushed to reduce the inclination to an acceptable value. In this process, a Teflon sliding plate was placed between the pier and the girder, so that the friction between the girder and the pier was very small, and can be ignored. Figure 26b shows that the excavation of the stress dissipation hole relieve the lateral pressure of the soil in the FEM model.

**Figure 27:**
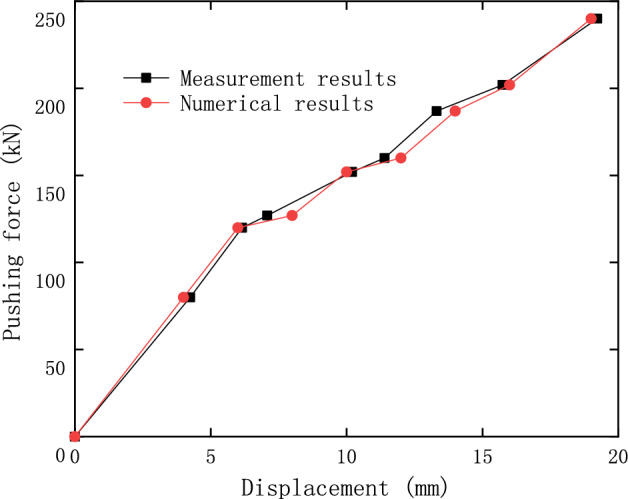
The displacement at the top of pier 22 in the lateral pushing rectification.

The relationship between the displacement and pushing load on the top of the pier is shown in Fig. 27, and it indicates that the numerical results can be used to predict the pushing force required for the pier rectification. When the pushing force is less than 120 kN, it has a linear relationship with the displacement, which indicates that the overall structure composed of pier, pile foundation and soil is in a linear elastic state. When the pushing force is greater than 120 kN, the elastoplastic deformation occurs in the soil, so the slope of the relationship curve between the pushing force and displacement becomes smaller and fluctuates.

**Figure 28:**
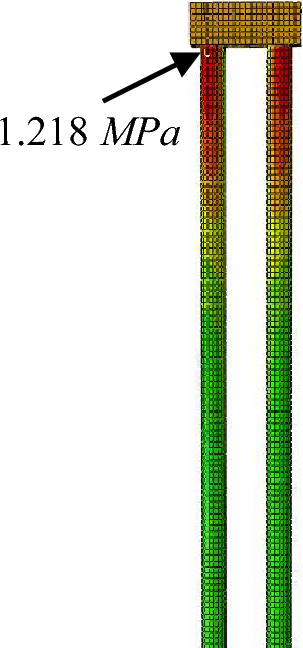
The maximum tensile stress in the piles below pier 22.

It was because that the top of pier 22 was pushed, the pier and the pile foundation below will bend. Therefore, on this basis, it is necessary to prevent the tensile stress in the pile from exceeding the allowable value, which might result in cracking damage. Figure 28 shows the calculated tensile stress of the pile foundation under pier 22. The maximum tensile stress, 1.218 MPa, at the top of the pile, is less than the tensile strength of concrete C25 used for the pile, 1.27 MPa. Therefore, on this basis, it can be concluded that the pile will not be damaged during the pushing.

## Conclusions

With an actual, physical accident as an example, the mechanism of inclination of the bridge structure in soft soil area under an adjacent surcharge load was analyzed, and rectification was proposed. The entire process of structural inclination caused by the surcharge, partial recovery caused by the unloading, and rectification by jack pushing were simulated numerically. On this basis, the effects of the underground soil properties, and the magnitude and loading time of the surcharge on the bridge inclination were studied. The conclusions are as follows:

1. The surcharge adjacent to the bridge will lead to the lateral pressure and displacement of the foundation soil, which will lead to the inclination of the bridge pile, the pier, and even the upper girder, resulting in bridge accidents.
2. In soft soil areas, the softer is the foundation soil, the greater is the surcharge load and the longer is the loading time, the greater is the inclination of the bridge structure caused by the adjacent surcharge load. Therefore, the more serious is the drainage consolidation of the foundation soil, the lesser is the inclination that can be reduced by unloading.
3. For the inclination of the bridge structure that cannot be reduced by the unloading, jack pushing can be used to rectify the inclination, in which the superstructure is recovered first by pushing the bridge girder, and then the substructure is corrected by pushing the pier.
4. For the bridge inclination accident caused by the surcharge, the numerical simulation can be used to predict the inclination by the surcharge and the recovery by the unloading, and evaluate whether the bridge structure is damaged, so that a foundation for the next step of structural correction and reinforcement may be laid.
5. For the bridge rectification by jack pushing, the numerical simulation can be used to calculate the pushing force required and the stress in the bridge structure, so as to determine the control value of the pushing force and prevent the additional structural damage during the rectification (Supplementary Information).

## Supplementary Information

Supplementary Information 1.

Supplementary Information 2.

## Data Availability

The datasets used and analysed during the current study available from the corresponding author on reasonable request.
