# Supplementary material for: A numerical analysis of inclination and rectification of ramp-bridge piers adjacent to surcharge load in soft clay area
Source: Sci Rep. 2023 Jun 14;13:9614. doi: 10.1038/s41598-023-36737-6 (PMC10267190; doi:10.1038/s41598-023-36737-6)
Supplement: Supplementary file 1 — Supplementary Information 1. [file 41598_2023_36737_MOESM1_ESM.doc]

Introduction of Supplementary Files

1. Model_and_subroutine_file_in_FEM_analysis.zip

This is a compressed file including two files used in the finite element software ABAQUS. The first file is ‘Model.cae’, which contains the computational model in this manuscript, including the geometric data of each component, material properties, element division, the interaction between components and the boundary condition information. It also contains the whole process of the simulation including loading and unloading of the earth heaps. The second file is ‘Subroutine.for’, that is a subroutine file called in finite element analysis in ABAQUS, including the sigini subroutine for balancing the ground stress and the voidri subroutine for defining the initial pore ratio.
